# Music, Rhythm and Trauma: A Critical Interpretive Synthesis of Research Literature

**DOI:** 10.3389/fpsyg.2020.00324

**Published:** 2020-02-27

**Authors:** Katrina Skewes McFerran, Hsin I. Cindy Lai, Wei-Han Chang, Daniela Acquaro, Tan Chyuan Chin, Helen Stokes, Alexander Hew Dale Crooke

**Affiliations:** ^1^Creative Arts and Music Therapy Research Unit, Faculty of Fine Arts and Music, The University of Melbourne, Melbourne, VIC, Australia; ^2^Melbourne School of Graduate Education, The University of Melbourne, Melbourne, VIC, Australia

**Keywords:** music, rhythm, trauma, adverse experiences, critical

## Abstract

Recent theorizing about the connection between the brain and trauma (Perry, 2009; Porges, 2011; van der Kolk, 2015) has led to a burgeoning of interest in the provision of music-based programs with people who have had adverse experiences. Although there has been critique of the lack of scientific basis of these theories and their implications for practice (McLean, 2016), they remain popular with practitioners who are keen to introduce innovative and potentially beneficial approaches to the people with whom they work. Music therapists have a long tradition of working with traumatized clients, however, the brain-based rationales did not seem congruent with the less predictable and more idiosyncratic benefits reported, which seem to occur through more psychodynamic mechanisms of action. In order to unravel what seemed to be a body of literature plagued by the conflation of theories, we undertook a critical interpretive synthesis of literature in the past 10 years to cross-examine the ways that music and trauma have been connected. To do this we extracted data from 36 identified articles to distinguish what music methods were used, what claims were made about benefits, what theoretical justifications were provided and how much research basis there was for the claims being made. Having systematically disentangled the various dimensions, we then constructed a spectrum of approaches that offers a logical categorization of four different ways of using music with people who have had adverse life experiences. These included using music for stabilizing, entrainment, expressive and performative purposes. Specific music-based methods were proposed for those associated with brain-based rationales, and more responsive, multi-method approaches were congruent with recovery and social change models. Future research would benefit from a more clearly articulated connection between theoretical rationale, music-based methods, benefits and research approaches. The resultant spectrum may provide useful guidance for both practice and research design.

## Rationale

International interest in the relationship between rhythm and trauma has burgeoned in response to recent endorsement through popular trauma discourse that relies on neurological mechanisms of action. A number of popular theorists' ideas have been used to suggest that rhythm-based activities are beneficial for people who have had adverse experiences because it bypasses higher cognitive functioning and allows connections to form via more primitive, undamaged regions of the brain. One of these theories has been posed by psychiatrist Perry (2009), who uses brain studies to argue that since trauma has a visible impact on the primitive brain, interventions which also operate at a pre-conscious level should logically be more effective than cognitively mediated strategies. Porges (2011) has developed a more complex theoretical explanation, labeled Polyvagal theory, to propose that trauma symptoms are mediated by an amygdala that has become hyper-vigilant to threat-related cues, and therefore activities which regulate physiological arousal will be helpful in stimulating the vagally modulated social engagement system in positive ways. van der Kolk et al. (2007) adds that rhythmic activities can fulfill this function by reawakening feelings of pleasure and engagement dulled by prolonged trauma exposure, and that rhythm stimulates patterned, repetitive neural brainstem activity necessary for restoration of brain functioning. These three men have had an enormous influence on music practitioners in the field of trauma, with an anecdotal increase in rhythm-based programs being noticed through social media posts and requests for information from music therapists by the public and by students and practitioners in trauma related fields.

Some critique has emerged of these perspectives and the practice implications that have results from them, often categorized as “trauma-informed” practices. McLean (2016) summarizes the precarious assumptions underpinning this discourse as follows.

- “The way in which brain development in the context of early adversity and trauma is represented may be oversimplifying the science;
- Claims regarding the plasticity of the brain and what it might mean for therapeutic intervention are not justified by the available science; and
- Therapeutic interventions that are based on these assumptions (e.g., song, rhythmic drumming, spinning), although popular, have not yet been subject to the systematic evaluation that other trauma-specific therapies have.” (p. 3).

We agree that there is a great deal of optimism in these theories and note that our practice experiences as therapists and teachers with young people who have had adverse childhood experiences suggests that the role of rhythm is less reliably mechanical, and more complexly woven into a fabric of relationship, musical encounters, creativity and safety. Because of our shared interest in the practical application of music/rhythm with young people, the final point made by McLean is our focus in this systematic review of the literature. Meticulous and systematic evaluation of these popular ideas is needed in order to ensure that they are effective, and prior to that, some of the key theoretical constructs need to be disentangled and clarified. To begin this process, we undertook a careful search of the literature that connects music with trauma, abuse and foster care. Our aim was to cross-examine the ways that music and trauma are connected in the literature. To do this, we extracted data to answer four questions from which we could draw conclusions in relation to the aim.

1. Which music methods are used by practitioners describing work with people who have experienced trauma, and to what degree is rhythm emphasized?
2. What claims are made about the benefits of using music to work with people who have experienced trauma?
3. What theoretical justifications are utilized for using music with people who have experienced trauma?
4. How much research basis is there for the claims being made?

## Method

### Critical Interpretive Synthesis

Systematic literature reviews have become an increasingly relevant method for scholars to interrogate a burgeoning literature that makes it difficult for readers to be fully cognizant of the breadth of theories and results that are constantly emerging. This notion was most popularly posed by Archie Cochrane, who recognized the divide between how clinicians were practicing in the field and the most recent findings being produced through medical research (Bero and Rennie, 1995). His intention was to increase awareness of findings in order to improve practice. The “Cochrane Reviews” then became a dominant and sometimes oppressive force within medical and educational practice (Aigen, 2015), accompanied by a demand for “evidence-based practice” which has been adopted by insurance companies and government agencies charged with distributing funds to service providers. The evidence-based movement is hierarchical, and privileges quantitative data over qualitative knowing because it supports the application of tenets of objectivism such as randomization and controlled conditions. The implications of this uneven emphasis have been critiqued, but this view continues to dominate in many cultures.

One alternative approach that has grown from the same desire to organize knowledge in ways that can inform readers about emerging ideas (Sackett et al., 1996), is the critical interpretive synthesis. Rather than summarizing knowledge, this critical approach recognizes that some knowledge is considered more credible than others, and that trends in the literature often emerge from a culture of privilege (Baines and Edwards, 2015). Therefore, rather than reinforce dominant ideas, a critical approach seeks to reveal the forces that have been previously rendered invisible, as articulated by Bourdieu (Navarro, 2006). There is an existing tradition of critical literature reviews such as these in the medical (Dixon-Woods et al., 2006) and ethnographic (Noblit and Hare, 1988) fields which provides a precedent for adopting a similar approach in the current investigation. By adopting a recursive but systematic approach it becomes possible to interrogate the literature, rather than to accept it, thereby challenging assumptions and recognizing forces at play that underpin knowledge generation (McFerran et al., 2017). This approach is well-suited to our aim of cross-examining the ways that music and trauma are connected in the literature.

### Search Strategy

We undertook a number of searches to identify relevant literature, including a combination of words for “music” + “trauma.” The alternate search terms used for music included: music, drum, drumming, hip hop, rhythm, improvisation, rap, beat making, songwriting, sing, singing, song, GIM (Guided Imagery and Music), and audio. Two additional terms were used as alternatives to trauma: abuse and foster care. Excluded topics were more focused on the event rather than the response and included: surgical trauma, medical trauma, cultural or historical trauma, immigration, political trauma, traumatic injury, physical trauma, traumatic event (not specifying the result of trauma), musician's trauma and substance abuse.

Our search focused on articles published in the last 10 years, which was from 2009 to 2018. Ninety-one sources were identified through this search, including a range of manuscript types. We decided to focus on peer-reviewed literature including journal articles and dissertations, and excluded chapters and books from the analysis, we also removed repetition of research projects that were reported in multiple articles. This reduced the number to 36 sources which are summarized in Table 1. Twenty-six of these were research studies and ten were case studies that did not ultimately qualify as research but which provided in-depth, detailed descriptions.

**Table 1:** Included studies.

**References**	**Title**	**Country of study**	**Search terms**
Alanne (2010)	Music psychotherapy with refugee survivors of torture: interpretations of three clinical case studies	Finland	Guided Imagery and Music + trauma
Beck et al. (2018)	Feasibility of trauma-focused Guided Imagery and Music with adult refugees diagnosed with PTSD: a pilot study	Denmark	Guided Imagery and Music + trauma
Bensimon et al. (2012)	A pendulum between trauma and life: group music therapy with post-traumatized soldiers	Israel	Music + trauma
Bensimon et al. (2017)	The emotional impact of national music on young and older adults differing in posttraumatic stress disorder symptoms	Israel	Music + PTSD
Blanaru et al. (2012)	The effects of music relaxation and muscle relaxation techniques on sleep quality and emotional measures among individuals with posttraumatic stress disorder	Israel	Guided Imagery and Music + trauma
Bolger (2015)	Being a player: understanding collaboration in participatory music projects with communities supporting marginalized young people	Australia	Songwriting + Out of home care
Carr et al. (2012)	Group music therapy for patients with persistent post-traumatic stress disorder - an exploratory randomized controlled trial with mixed methods evaluation	U.K.	Music + trauma
Christenbury (2017)	I will follow you: the combined use of songwriting and art to promote healing in a child who has been traumatized	U.S.A.	Songwriting + abuse
Colegrove et al. (2018)	Pilot randomized controlled trial of Tuning Relationships with Music: intervention for parents with a trauma history and their adolescent	Australia	Music + trauma
Day et al. (2009)	Experiences of song writing in a group programme for mothers who had experienced childhood abuse	Australia	Songwriting + trauma
der Heyde and Christine (2012)	Interpersonal rhythms disrupted by a history of trauma: an in-depth case study of analytical music therapy	U.S.A.	Improvisation + trauma
Fairchild (2018)	Collaborative songwriting with children experiencing homelessness and family violence to understand their resources	Australia	Songwriting + abuse
Faulkner (2017)	Rhythm2Recovery: a model of practice combining rhythmic music with cognitive reflection for social and emotional health within trauma recovery	Australia	Music + trauma
Felsenstein (2013)	From uprooting to replanting: on post-trauma group music therapy for pre-school children	Israel	Music + trauma
Flores (2013)	African drumming as a medium to promote emotional and social well-being of children aged 7 to 12 in residential care		Drum + foster care
Gerber et al. (2014)	Children after war: a novel approach to promoting resilience through music	U.S.A.	Music + PTSD
Graham (2011)	Effect of music therapy on the emotional expressivity of children and adolescents who have experienced abuse or neglect	U.S.A.	Songwriting + abuse
Greene et al. (2009)	The use of expressive therapies and social support with youth in foster care: the performing arts troupe	U.S.A.	Drum + foster care
Hannigan and McBride (2011)	Drumming with intimate partner violence clients: getting into the beat; therapists' views on the use of drumming in family violence treatment groups	Canada	Drumming + trauma
Hunter and Rosevear (2011)	Evaluating a creative arts program designed for children who have been sexually abused	Australia	Drum + Foster care
Jespersen and Vuust (2012b)	The effect of relaxation music listening on sleep quality in traumatized refugees: a pilot study	Denmark	Music + trauma
Jespersen and Vuust (2012a)	Music for improvement of trauma-related sleep problems	Denmark	Music + trauma
Kim (2013)	Music therapy with children who have been exposed to ongoing child abuse and poverty: a pilot study	South Korea	Music + abuse
Neupane and Taylor (2011)	Music therapy for incarcerated women recovering from trauma and abuse	U.S.A.	Music + trauma
Osborne (2012)	Neuroscience and “real world” practice: music as a therapeutic resource for children in zones of conflict	U.K.	Music + PTSD
Palidofsky and Stolbach (2012)	Dramatic healing: the evolution of a trauma-informed musical theater program for incarcerated girls	U.S.A.	Songs + trauma
Precin (2011)	Occupation as therapy for trauma recovery: a case study	U.S.A.	Songs + trauma
Reeves (2013)	How music and lyrics protect and heal the souls of African women who have experienced domestic-violence trauma, sexual abuse, or depression: a phenomenological study		Music + trauma
Rudstam et al. (2017)	Trauma-focused group music and imagery with women suffering from PTSD/complex PTSD: a feasibility study	Sweden	Music + trauma
Salmon and Rickaby (2014)	City of one: a qualitative study examining the participation of young people in care in a theater and music initiative	U.K.	Music + foster care
Schrader and Wendland (2012)	Music therapy programming at an aftercare center in cambodia for survivors of child sexual exploitation and rape and their caregivers	U.S.A.	Songwriting + trauma
Story and Beck (2017)	Guided Imagery and Music with female military veterans: an intervention development study	Denmark	Music + PTSD
Strehlow (2009)	The use of music therapy in treating sexually abused children	Germany	Music + trauma
Sutton and De Backer (2009)	Music, trauma and silence: the state of the art	U.K. and Belgium	Music + trauma
Wellman and Pinkerton (2015)	The development of a music therapy protocol: a music 4 life® case report of a veteran with PTSD	U.S.A.	Drumming + trauma
Zanders (2015)	Music therapy practices and processes with foster-care youth: formulating an approach to clinical work	U.S.A.	Music + foster care

### Data Extraction

Once the articles were identified, data was extracted from each against a set of questions as reported above. The intention was to generate a concise answer to the question, and not to rely on direct quotations which could require entire paragraphs. In some cases, interpretation was required if the information was not explicit. For example, not all authors distinguished between aims and outcomes, or gave details about the specific music method being used. The research assistant worked closely with the primary author to determine answers in these cases, using a combination of logical and reflexive questioning to determine if the information could be interpreted or if doing so was beyond our abilities to assume. For example, limited information about the setting of the program might be supplemented through online searching, or making assumptions based on the language used to describe the participants which may be more typical of medical or community contexts. We relied on the information published and did not make contact with authors if data was missing. The data were extracted in answer to the following prompts.

- How the participants are described—e.g., their behaviors and their presenting issues
- How the participants' trauma is described/diagnosed—e.g., complex, PTSD, etc.
- The reported cause of the trauma—e.g., events
- Age
- Setting
- Race/ ethnicity
- Group or individual program
- Number of people
- Research or not? If so, chosen design
- Claims about program aims
- Claims about program findings/results
- Music methods used
- Music genres referenced
- Any specific references to rhythm
- Citations used as justification for using music with traumatized people.

### Data Analysis

Simple descriptive analysis was used to analyze the data in regard to the four research questions as a beginning point. This process required some interpretation when answers were not readily available, but our intention was to remain close to what the authors were describing in their articles, defined by our extractions being “recognizable.” The over-arching question about how music and trauma are connected within the literature was then examined via meta-synthesis—exploring patterns that presented across the data, often examined through the intersections between multiple categories. This was undertaken with particular attention to critical questions about assumptions underpinning decisions, what was missing as well as what was dominant, who seems to benefit from the findings, and also attending to any emotional responses (frustration, sadness, surprise) to emerging patterns (McFerran et al., 2017).

## Findings

### Music Methods

Music has a long history of application in the mental health field and practitioners have been using an array of music-based methods with people who have had adverse life experiences for many decades (e.g., Rogers, 1993). For example, the profession of music therapy was established in the 1950s in the U.S.A. in response to the high number of veterans who returned from fighting in the war with post-traumatic stress disorder, as well as in response to later wars (Slotoroff, 1994). Some of the authors are qualified music therapists, and therefore have a particular interest in, and knowledge of this discourse. This potentially influenced the balance of articles with just under half being music therapy articles (*n* = 17), 9 being from psychology, and 10 coming from other disciplines including other creative arts and expressive arts therapies, guided imagery and music, education, occupational therapy, nursing and arts backgrounds. In categorizing the methods used however, we have not distinguished between music therapists uses of particular methods, such as song writing.

**Figure 1:**
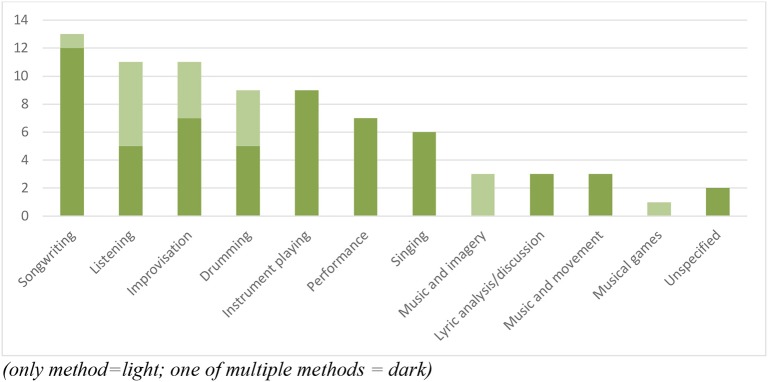
Frequency of reference to music-based methods (only method = light; one of multiple methods = dark).

We began our investigation expecting to find an emphasis on rhythm in the literature given the prominence of reference to this musical element in the social discourse emerging from the neurological theories about trauma. In the initial search, the term “rhythm” did not result in any identified sources, nor did “beat making” or “audio.” We reviewed our conception of “rhythm” and chose to expand our searching to recognize that rhythm is inherent in music and that we were interested in the degree to which it was made prominent by different authors. As noted above, we then used 14 terms for music in our searches of the peer-reviewed literature of which rhythm was the primary focus of 5 of the 37 manuscripts identified, with group drumming being the sole method described in all but one of these. In addition to the four using group drumming exclusively, 9 of the 37 articles in total included drumming as one of their methods and it was the fourth most common method described along with those that described playing on other instruments. Songwriting was the most common method referenced and both music listening and the music therapy technique of improvisation was reported in 11 articles. There was also reference to performances, singing, and others represented in Figure 1.

In further interrogating the representation of different methods within the literature, it became clear that there was an even split between those focusing on the use of one method only and those using multiple methods, with 18 manuscripts describing between 2 and 6 methods (average 3.6). Of those focusing on a single method, half relied on receptive music experiences, including music listening for analysis of lyrics, imagery, relaxation, and other meaning making processes that relied on associations being explored.

Although we only focused on literature in the past 10 years, we were interested to examine how many different approaches were being described in contemporary discourse, and to consider what trends may have impacted these findings. Only one study made reference to Hip Hop, despite our combined experience suggesting that it is a popular method in current practice, particularly in the provision of services for youth. This suggests that the research is lagging behind practice in this space, as McLean (2016) has suggested. It would be a mistake to assume this means Hip Hop is not relevant and/or helpful, however. A strong theoretical rationale exists for the value of culturally relevant music practices (Stige, 2002), whether that is related to black American culture, or youth culture more generally. Similarly, the prominence of songwriting does not imply this method is most useful, but rather, that it is most published.

### Theoretical Basis

**Figure 2:**
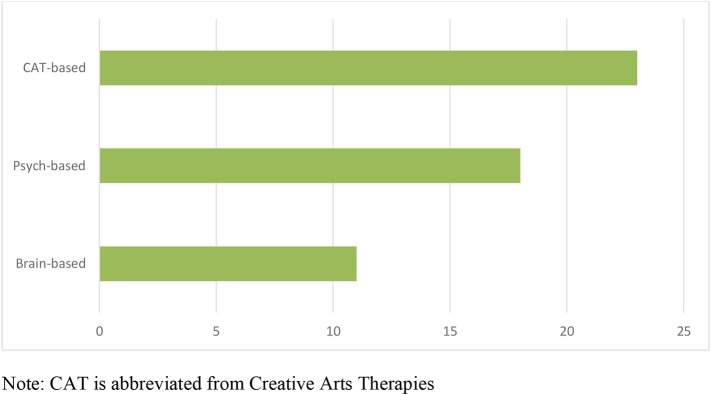
Theoretical rationale provided in included manuscripts. CAT is abbreviated from Creative Arts Therapies.

One important part of the rationale for this investigation was the influx of interest in the connection between music, trauma and the brain. In our view, this has largely been at the instigation of three popular authorities in the field of trauma—Perry (2009), van der Kolk et al. (2007), and Porges (2011). In addition, music therapy theorist, Thaut (2005), has long posited that the benefits of music therapy are best explained by studying brain based reactions, and has been particularly interested in the role of rhythm in this context. Our first interrogation of the theoretical underpinnings of the 37 studies was therefore to determine the degree to which a brain-based rationale was provided for music-based interventions (see Figure 2). Ten of the articles made reference to either Porges or van der Kolk, with van der Kolk being the most referenced theorist (8 articles), while Porges was only noted in three and Thaut in one. The second most prominent theorist was Herman (2015), whose work on “Trauma and Recovery” transformed the discourse in the early 1990s and is probably still the most influential trauma text of our time. Herman does not directly address music, but rather it is her three-stage model of recovery that many practitioners use as a guide to their work. Perry was not referenced in any of the articles, perhaps because he is more well-known as a presenter than a published author.

Music therapy theory and theory from other Creative Arts Therapies (CAT), including the specific field of Guided Imagery and Music which requires advanced training, was also referenced frequently in the included manuscripts, once again reflecting the professional training of some of the authors. No single CAT authors were referenced in particular, and many music therapists also integrate theoretical approaches dominant in psychology and psychiatry. This was true of the rationales used across many articles, including psychoanalytic and psychodynamic theorists, cognitive behavioral therapy, attachment theory, gestalt theory, as well as some scholars from music psychology and creative arts therapies more broadly. Resilience was referenced by a significant number of authors, although no particular resilience theorists were dominant.

### Ascribed Benefits

Two sets of data were included in this analysis. The primary data was the findings/results that were described for participants in the music-based programs. However, we also felt it was important to critically analyze the authors' aims, since assumptions are often embedded in the intentions for a project, and therefore interrogating them may be revealing. This was certainly the case in our investigation, with 22 of the authors describing aims that included demonstrating how music could serve as a useful medium for people who had adverse experiences. This focus reveals a high degree of investment in positive outcomes; however, minimal reflexivity was presented in the qualitative research, and only two of the quantitative studies included a control condition so that an objective comparison could be made. Either of these approaches to confirming how findings may have been influenced by the expectations of the researchers would have enhanced the quality of the literature. Whilst it is natural that practitioner-researchers should believe in their approach, and certainly, it would make no sense to investigate something considered to be unhelpful, scientific standards demand that some effort is therefore made to ensure findings are trustworthy. Further development is needed in this area.

Working in the field of trauma is complex and unlike some other fields of practice where music is used (for example, rehabilitation), specific improvements cannot always be readily targeted and measured. It can be difficult for people with lived experience, their carers, and their workers to know exactly what part of the complex psychological ramifications of adverse experiences should be focused upon—behavior, memories, meaning-making, and emotions are all intertwined and difficult to separate. Herman (2015) popular model recognizes this by focusing on different program aims for each stage rather than focusing on benefits or expected outcomes. She does not make generalizable predictions about universal benefits but focuses on recovery as a unique and ongoing journey. However, there is a strong (reductive) trend in the cognitive and psychological sciences more generally that brain-based explanations appear to solve because it provides elegant explanations that could explain the complexity. However, the reality of the complex terrain of living with trauma is reflected in the kinds of benefits reported by authors in the literature. A focus on intrusive trauma symptoms that could be paired with measurable outcomes appeared adequate only for those living with the most severe behavioral responses. For example, *symptom reduction* was a target in 11 manuscripts of which seven were describing trauma experienced in military contexts, and six of this sub-set used quantitative designs and two used mixed methods. Another behavioral outcome noted in the analysis was a focus was on *sleep and/or relaxation*, represented in five articles.

However, for those with adverse life experiences beyond the military it seemed more difficult for authors to nominate specific benefits and there was a great deal of variety in the ways that authors couched the outcomes they were reporting. For example, an emphasis on *resilience* and/or *empowerment* was present in five articles that were set in community-based contexts, including a kindergarten and youth center. *Social* benefits were posited in a different set of seven manuscripts, and all of these were related to programs provided in groups, rather than individual therapy where trust in the therapist might be more relevant, although not usually described as a benefit. *Self-worth* was another important benefit posited in a different set of eight manuscripts, with all of these being programs for marginalized young people in non-acute contexts. Some kind of *emotional* outcome was referenced by eight of the authors, but these were difficult to categorize as benefits, and were mostly described more generally as having opportunities for emotional expression and processing rather than improvements *per se* (i.e., emotional responsiveness, emotional balance, emotional expression, work through emotional aspects, understanding of emotion, expressing musical emotions, regulate and express emotions). The term affect was used by some quantitative researchers (i.e., affect and mood stabilization, fewer affective and cognitive disturbances), and feelings were also referenced by some authors (express repressed feelings, increased expression of non-traumatic feelings). Although the benefit of working with emotions was difficult to articulate, emotional challenges are central to many traumatized peoples' experience and the opportunity to participate seems justifiable as a benefit of therapeutic work with music.

There was a suite of 11 articles in this review from which it was more difficult to extrapolate specific well-being benefits to participants and that were more exploratory in nature. Many of these were focused on exploring whether music-based programs were useful in the diverse contexts where they had been introduced, from inpatient and outpatient mental health programs, private practice, and a range of community-based programs for survivors of torture, sexual exploitation, refugee detention and those in the foster care or family violence systems. Authors described aims such as:

1. Exploring the ways in which music can speak directly to the trauma, and how music therapy offers a unique means to understand the traumatized patient
2. Exploring whether and how rhythmic interactions within musical improvisations facilitate the repair of ruptures in such rhythms
3. Describing women's experiences of song writing to support parenting
4. Describing the evolution of an innovative program for incarcerated adolescent girls in which youth work collaboratively with theater professionals to create, develop, and perform musicals based on their experiences
5. Understanding to what extent, and in what ways, the mental health condition of the research subjects changes during music psychotherapy treatment
6. Exploring the ways in which music therapy programming can support and enhance care systems already in place for survivors of sex trafficking and their care staff
7. Presenting a new way of combining songwriting and visual art to facilitate emotional expression
8. Balancing the representation of children in this context by using music to explore their resources and what helps them to “do well” throughout their experiences of homelessness and family violence
9. Presenting a framework for music therapy processes and practices with foster-care youth
10. Describing how a performing arts troupe program supports vulnerable youth
11. Helping to develop musical aspects of a local program that contributes to the welfare of children and to use the social power of music to raise awareness of their situation in a wider world.

### Research Approaches

**Figure 3:**
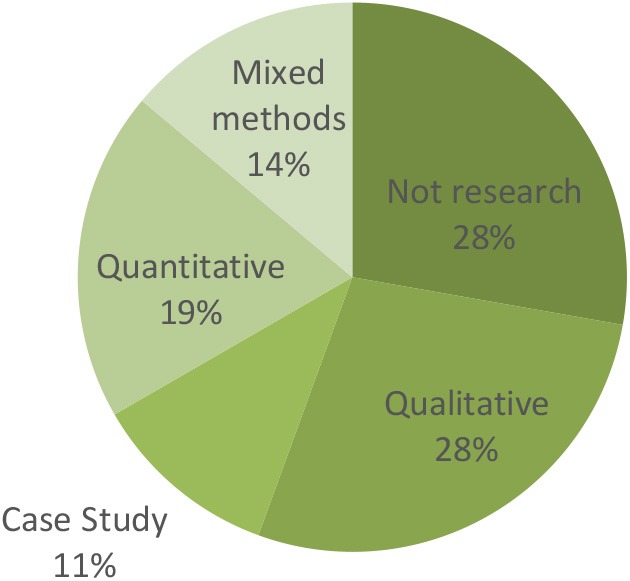
Research approaches used in 26 manuscripts.

This critical interpretive synthesis does not aim to synthesize all the literature on the topic of rhythm and trauma, but rather, to interrogate the ways that music and trauma are connected in the peer reviewed literature. Our decision to focus on peer-reviewed literature was intentional and we aimed to avoid the inclusion of anecdotal report and to focus on what McLean (2016) calls science, but what we would call research-based reports. Despite this intention, of the 36 manuscripts included in our investigation, 10 were not research articles but were descriptive program reports without a clear research methodology. They did contain useful information in answer to the remaining questions however, and, were included in the analysis of other sections. Of the 26 manuscripts that had a clear methodology, the research approaches were dispersed across data types, with qualitative research being most common, and although the case studies are displayed separately, these all used qualitative approaches. Those studies that used quantitative data included two randomized controlled trials, while the rest of the studies did not have control conditions or randomization (see Figure 3).

The studies relying on quantitative data came from a range of regions, including Israel (3) and the Middle East (2), U.K. and Denmark, as well as Australia and the U.S.A. The qualitative studies were more likely to come from Australia (4) or the U.S.A. (3), with one each from the U.K. and Denmark, and the case studies were similarly dominated by north American authors, with one set of three case studies from Finland. There are a number of possible explanations for the limited number of objectivist studies. It may suggest that the interface between trauma and music is considered to be challenging by many to quantify, or that ethics approval for conducting studies with people considered to be so vulnerable is difficult in western countries with stringent systems for monitoring and approving research projects. Pragmatically, it can be difficult to adequately power experimental research studies with this population due to limited sample sizes. Alternatively, it might be that authors' felt quantitative data was incongruent with the importance of narrative that is often emphasized in therapeutic works with people who have had adverse experiences.

## Meta Synthesis

The notion of meta-analysis in quantitative systematic review refers to the use of statistical methods to combine the data from a large number of studies. In qualitative syntheses, the “meta” does not refer to aggregating, but rather to broadening understanding of a particular phenomenon (Grant and Booth, 2009), in this case through identifying more nuanced and qualitative distinctions between strands of data. After immersion in the results of the analysis above, we constructed a spectrum of approaches that could be used to distinguish four categories aligning with a set of proposed musical purposes relevant for working with people who have lived experience of trauma (see Table 2). The spectrum transpired after grappling with how theories that posit primitive neural activity as key mechanisms of action (such as Porges, 2011; van der Kolk, 2015) were related to specific types of music methods suggested and this led us to conceptualize the first two categories. What remained were approaches that required higher level brain activity such as emotion processing and social awareness and these were then connected to different music methods proposed. Benefits were also an important consideration in constructing the emerging spectrum, since we were endeavoring to untangle various strands of discourse in order to identify whether there could be cogent links between theorized mechanisms of action, music methods and expected benefits. We used abductive logic to explore this possibility in the meta-synthesis and as a result, speculate that there are four ways of understanding how music and rhythm can afford a range of benefits for those who have had adverse life experiences resulting in trauma.

**Table 2:** Spectrum of approaches to music and trauma.

**Musical purpose**	**Stabilizing**	**Entrainment**	**Exploratory**	**Performative**
Music methods	Focused music listening	Group drumming, structured improvisation, shared singing	Music psychotherapy—improvisation, guided imagery, song writing	Song writing and performance
Neural activity	Primitive	Primitive	Cognitive	Multiple
Relevant theories	Porges, Perry	Siegel, Ogden	Herman, Van der Kolk	Herman, Butler
Form	Individual	Group	Individual or Group	Group
Context	Short-term acute	Community	Long-term recovery	Community
Therapeutic focus	PTSD symptoms	Dissociation	Integration	Recovery
Regulatory	Physiological regulation	Co-regulation	Emotion regulation	Self-regulation
Reported benefits	Reduced negative symptoms, sleep, relaxation	Varied	Emotional work, self-worth	Social connections, resilience
Research	Quant	Varied	Varied	Participatory/Program description

### Stabilizing

The term stabilizing has been used to label this group of manuscripts because there was no suggestion of the music being used in a responsive way. Rather, listening to recorded music was the selected method for individuals with a PTSD diagnosis because it provided a safe structure affording a stable pulse lasting for the duration of each musical piece. This allows for predictability when used in a repeated way and emphasizes tempo rather than rhythm *per se*. Importantly, recorded music is reliable as a variable within quantitative research designs and music listening was found to be effective in influencing physiological regulation, resulting in improved sleep, increased relaxation and the reduction of negative PTSD symptoms.

The theoretical basis for this category relies on the premise that physiological processes can be modulated via brain-based processing that bypass areas of the brain damaged by trauma. For example, Perry's neurosequential model (Perry, 2006, 2009) suggests that regular and repetitive rhythmic activities can regulate these primitive areas of the brain, even when early adversity has led to chronic hyper-arousal. Porges' theorizing also implicates rhythm as a way to calm an over-sensitive central nervous system by drawing on the autonomic nervous system (Porges, 2011). He introduced the term neuroception (Porges, 2004) to refer to the ways the autonomic nervous system evaluates risk in the environment, and suggests that prosodic acoustic stimulation signals safety. These theories are precisely the ones criticized by McLean (2016) because of the lack of scientific evidence. Whilst we believe her critique is well-founded, these studies do demonstrate that music listening can improve physiological markers of distress in people who have had adverse experiences. These are likely to be temporary, rather than leading to healing in the brain, as suggested by these popular theorists, however they may still be appreciated by those benefiting from greater amounts of sleep and relaxation.

### Entrainment

The term entrainment has a long history in musicological and music psychology discourse and is usually paired with the notion of synchronization. Some scholars distinguish entrainment as the purpose and as “a process that governs the alignment of the auditory and motor domain,” whilst synchronization is the action, being “a dynamic attraction point where the timing differences between music and person are stabilized” (Moumdjian et al., 2018). In the music psychology literature, it is often about pairing human behavior with an external auditory source, as described by Cameron and Grahn (2016): “The synchronization of internal rhythm processes (such as neuronal oscillations) or behavior (such as tapping or dancing) to external, periodic events (e.g., the beats in a rhythm)” (p. 363). However, in the ethnomusicology literature, the freedom to oscillate rather than be mechanically steady is emphasized (Clayton et al., 2005), which is critical to its use in this meta-synthesis since keeping perfectly in time is neither necessary nor likely when working with non-musicians.

The term entrainment has been chosen here because it is a logical extension of an approach that relies on primitive neural activity as a mechanism for bypassing more complex trauma related behaviors such as hyper- and hypo-arousal—bringing people into a more optimal “window of tolerance,” as described by Siegel (1999). This kind of sensorimotor approach has been advocated by much quoted practitioner-researcher, Pat Ogden (Ogden et al., 2006), who suggests connecting with people via basic functions such as rhythm when cognitive processing and self-regulation are considered to be unavailable. Hence music methods that rely on clearly guided musical experiences emphasizing basic pulses and synchronized activity are a good match for this theorizing.

Drumming is the most obvious music method that can be classified as a sensorimotor activity in music with basic research suggesting that this should result in improvements through co-regulation. Drumming groups often imply high levels of structure and rule-following, as distinct from the more emergent and responsive approaches described in the Expressive category. However, drumming appears to have been integrated into a range of approaches in the literature and used in both dynamics and more ordered ways. This distinction is critical for facilitators to consider, since the way drumming is used would logically lead to quite different outcomes. This point has been made in Michael Thaut's rhythm-based approach to music therapy (Thaut et al., 2015) which has been tested in rehabilitative contexts, but for which there is less evidence in mental health domains. Critical to this approach is focused and repetitive music activities that are reliably delivered with strict adherence to musical parameters, similar to a behavioral approach. This does involve live music making, so that the therapist can make adjustments to timing where necessary, however, it is very different from a psychodynamic approach that may use drumming experiences as the basis of interpretation and fostering insight. In the literature reviewed, it was not always possible to understand how rhythm-based methods were being offered.

### Expressive

This category takes expression in its title because it encompasses an array of personally expressive dimensions—including emotions and feelings as one component, but also the broader constructs of personal expression, identity work, and a range of other well-being benefits. Rather than bypassing damaged regions of the brain, as posed in the previous two categories, the expressive emphasis is about acknowledgment and integration of adverse experiences. This requires higher cognitive functioning and the ability to actively reflect and make meaning and is classically associated with psychotherapy, where uncovering repressed experiences is considered critical to recovery. However, following on from the seminal work of Herman (2015), contemporary trauma work emphasizes the importance of establishing safety and stability before progressing to reconstructing the trauma narrative. This may explain the mixture of methods used in this category, where song writing can be used for reconstructing the trauma narrative, and improvisation for exploring less conscious reactions, and movement through a range of other activities can provide opportunities for moving back into relationship building and re-establishing safety.

van der Kolk's (2015) work provides a good theoretical framework for this category, with an emphasis on finding ways to be fully alive and engaged. Self-exploration within a safe therapeutically contained relationship is critical to this grouping, which is all contributed by trained professional psychotherapists specializing in music—music therapists. Carefully managed, expressive arts-based activities can be useful in re-establishing ownership of body and mind (van der Kolk et al., 2007) through becoming more familiar with ways of expressing the self—one's history, aspirations, relational capacities and hopes and dreams. This incorporates the emotional work noted by Herman (2015) in the second stage of remembrance and mourning, and having multiple creative methods available means that the therapist can circle back into safety and self-soothing, or forward into reconnection as required.

### Performative

The distinction between personal work and recognition that war and abuse occur in a social context that condones or tolerates these adverse experiences is critical to the distinction between this and the previous category. The word performative has been selected because it encompasses this critical positioning [influenced strongly by Butler (2010)], where identity is bought to life through words and actions rather than being an expression of something that is already fixed. This upends the notion that people's lives have been determined by their adverse experiences, and focuses on how certain affordances invite agency (Withagen et al., 2017), in this case, how public, creative affordances offered through musical performances can invite reconstruction of identity and also, advocate for social change.

As a critical scholar, Herman's (2015) final stage of recovery is aligned with the integration of private and public self that is demanded by the performative category. Other renowned critical scholars from outside the trauma field also provide an important framework for understanding. Freire's (1993) pedagogical theories for education are often applied in community, and emphasizes the importance of political performative acts to bring about social change. Similarly, arts-based approaches have been theorized with social justice agendas, such as Community Music Therapy (Stige et al., 2010) and Community Music more broadly (Bartleet and Higgins, 2018). Although not specifically theorized for those who have had adverse life experiences, these discourses do have oppressed and marginalized persons at their center, with an emphasis on society's responsibilities for the conditions that allowed abuse, rather than centralizing the individual's experiences of it and certainly not the neural pathways in the brain.

This category is therefore not restricted to contributions by qualified therapists, but also includes community artists, public health researchers, and other allied health practitioners working in community contexts. Groups are privileged because of the opportunities they afford for internal as well as public-facing performances, and the benefits are linked to these social possibilities. Interestingly, they are reported most often by privileged practitioners in first world economies working with people from marginalized backgrounds, including people traumatized through experiences of war in other countries.

## Conclusion

This critical interpretive synthesis grew from a shared frustration with a set of literature that seemed to conflate a range of theories about trauma and music-based practices. Ideas about how rhythm influences brain activity were used as a rationale for a wide range of music-based experiences that placed little emphasis on rhythm. The possibilities of bypassing damaged areas of the brain through musical engagement seemed to draw attention away from critical therapeutic processes built through safety and containment. Beliefs about what music could do to the brain seemed to override fundamental therapeutic tenets such as responsiveness, compassion and careful exploration of repressed material through music. This enthusiasm for a scientific rationale seemed to bear little relevance to our shared experiences of working with young people who had adverse childhood experiences as teachers, therapists and researchers.

By first asking specific questions of the literature and extracting data into discrete categories, we were able to identify where patterns existed and where no patterns could be discerned. For example, the theoretical rationales provided for most programs did not bear direct relevance to the specific type of music-based methods being used. We hope that this synthesis will encourage authors to articulate more clear lines of argument in the future. In addition, it became evident that there were high levels of bias in the research of all types, with little reflexivity, use of control groups, or any other strategies that might support a more scientific assertion of why music might be helpful for traumatized individuals and groups. Sometimes this was overt, such as when people's explicit intentions were to demonstrate how valuable their program had been, and other times it was covert but still missing. There was also little explanation for why specific music methods were used or how that decision related to the intended outcome. The inclusion of this information would benefit readers and scientists alike.

In order to make the discourse more amenable to scientific standards, a more coherent approach may be helpful that involves congruent connections between intentions, rationales, music methods, benefits and research methodologies. This is relatively straight forward when designing programs that target entrainment or stabilization—specific music methods should be linked to predictable outcomes if it is proposed that they will be activated through neural mechanisms, and these can be measured and compared to control conditions. Michael Thaut has modeled this approach in rehabilitation (2015), and appropriately advocates for strict application in order for this to be effective. By contrast, programs that have expressive or performative purposes do not rely on understanding music as a variable but instead draw more on sociological notions of music affording possibilities for constructing self-understandings and enacting social critique. In this context, multiple methods may lead to benefits at different times depending on what is needed by the individual of group in a given moment, with particular attention being paid to safety in the context of trauma. Subjective and emancipatory research is more congruent with these values and would benefit the field. One approach that might address the need for greater scientific basis would be to systematically compare different approaches to determine if there are differences in outcomes, which would confirm the necessary connections between inputs and outcomes. This would suit the first two categories of stabilization and entrainment, but might not capture the complexity of the second two, which would demand the inclusion of more subjective views collected through qualitative data, reflexively analyzed.

In concluding, it is important to recognize the complexity of the lived experience of trauma. For people to have presented to services for support that are then documented in the manuscripts analyzed, we can assume that adverse experiences have had a profound experience on their lives. There was clearly a need for helpful, and perhaps evidence-based support. However, given the statistics available at a population level on the high level of abuse that is perpetrated in most societies, we must also recognize that the response to adverse experiences is idiosyncratic, not universal. Some people survive without need for expert support, some people develop inspiring resilience and thrive in the same society that allowed abuse to occur. Therefore, it is logical that not all people benefit from the same supports, for a range of reasons including personal readiness, trust in therapist and services, suitability of services etc. A spectrum of approaches is therefore necessary, and it would be inappropriate to advocate for one over the other, but rather to attend to the individual in context and determine what music-based approach would meet their needs and desires at a given moment in time.

## Data Availability Statement

The datasets generated for this study are available on request to the corresponding author.

## Author Contributions

KM conceived the project, conducted the analysis, and drafted the article. W-HC undertook the initial data extraction, supported by HL. AC contributed to conceptualization and offered feedback. HS, DA, and TC contributed to analysis and editing.

### Conflict of Interest

The authors declare that the research was conducted in the absence of any commercial or financial relationships that could be construed as a potential conflict of interest.
